# Brain and Pituitary Response to Vaccination in Gilthead Seabream (*Sparus aurata* L.)

**DOI:** 10.3389/fphys.2019.00717

**Published:** 2019-06-18

**Authors:** X. H. Liu, A. R. Khansari, M. Teles, G. Martínez-Rodríguez, Y. G. Zhang, J. M. Mancera, F. E. Reyes-López, L. Tort

**Affiliations:** ^1^Department of Cell Biology, Physiology and Immunology, Universitat Autonoma de Barcelona, Barcelona, Spain; ^2^Key Laboratory of Freshwater Fish Reproduction and Development (Ministry of Education), Key Laboratory of Aquatic Science of Chongqing, School of Life Sciences, Southwest University, Chongqing, China; ^3^Instituto de Ciencias Marinas de Andalucia, Cádiz, Spain; ^4^Department of Biology, Faculty of Marine and Environmental Sciences, Instituto Universitario de Investigación Marina (INMAR), Campus de Excelencia Internacional del Mar (CEI-MAR), University of Cádiz, Cádiz, Spain

**Keywords:** brain, pituitary, vaccination, immune response, stress response

## Abstract

Vaccination is a widely used therapeutical strategy in aquaculture, but whether vaccination elicits stress responses in the central neuroendocrine system and enhances the crosstalk between the immune and endocrine systems in the brain or pituitary after vaccination is unclear. To answer this question two experiments using two different vaccine exposure routes, i.e., bath or intraperitoneal (i.p.) injection, were carried out on gilthead seabream (*Sparus aurata* L.). In the first one, the stress responses of fish subjected to waterborne *Vibrio anguillarum* bacterin were compared with responses after air exposure or their combination. In the second experiment, fish were subjected to an intraperitoneal injection of *Lactococcus garvieae* bacterin and we assessed the central stress response and also whether or not a significant immune response was induced in brain and pituitary. In both experiments, blood, brain and pituitary tissues were collected at 1, 6, and 24 h post stress for plasma hormone determination and gene expression analysis, respectively. Results indicated that bath vaccination induced a decreased central stress response compared to air exposure which stimulated both brain and pituitary stress genes. In the second experiment, injection vaccination kept unchanged plasma stress hormones except cortisol that raised at 6 and 24 h. In agreement, non-significant or slight changes on the transcription of stress-related genes were recorded, including the hormone genes of the hypothalamic pituitary interrenal (HPI) axis and other stress markers such as *hsp70*, *hsp90*, and *mt* genes in either brain or pituitary. Significant changes were observed, however, in *crhbp* and *gr*. In this second experiment the immune genes *il1β*, *cox2*, and *lys*, showed a strong expression in both brain and pituitary after vaccination, notably *il1β* which showed more than 10 fold raise. Overall, vaccination procedures, although showing a cortisol response, did not induce other major stress response in brain or pituitary, regardless the administration route. Other than main changes, the alteration of *crhbp* and *gr* suggests that these genes could play a relevant role in the feedback regulation of HPI axis after vaccination. In addition, from the results obtained in this work, it is also demonstrated that the immune system maintains a high activity in both brain and pituitary after vaccine injection.

## Introduction

Stress is defined as a state of real or perceived challenge for homeostasis that induces a response consisting in an array of biological reactions to compensate for the consequences of the threat created by the stressor (Tort and Teles, 2011; Schreck and Tort, 2016). After the stressor is perceived, the neuroendocrine cells of the ventral parvocellular section of the nucleus preopticus, secrete different neuroendocrine players: Corticotropin Releasing Hormone (CRH), CRH Binding Peptide (CRHBP), Arginin Vasotoccin (AVT) and Thyroid Releasing Hormone (TRH) that control the production of adrenocorticotropic hormone (ACTH) in corticotropic cells of the anterior pituitary gland (Flik et al., 2006). The release of ACTH into the bloodstream and interaction with the receptors of interrenal tissue, will subsequently induce cortisol release (Gorissen and Flik, 2016). Cortisol acts as a multifunctional hormone via binding to its receptors, the mineralocorticoid receptor (MR) and glucocorticoid receptors (GR), which are ubiquitously expressed in almost all tissues (Sapolsky et al., 2000; Teles et al., 2013). During the stress response, cortisol will redirect energy utilization among various organs in order to overcome the increased metabolic demand imposed by the stressor challenge. As a consequence, some processes such as immune response mechanisms may be affected or delayed (Kaattari and Tripp, 1987; Padgett and Glaser, 2003; Kudielka and Kirschbaum, 2007). For instance, hypothalamic CRH may act as an anti-inflammatory via stimulation of glucocorticoids and catecholamines; peripheral CRH acts as pro-inflammatory through direct action on immune cells (Karalis et al., 1997; Quintanar and Guzman-Soto, 2013) and cortisol acts generally as immunosuppressor or immunomodulator (Tort, 2011). Besides, ACTH has been reported to present immunoreactive activity in the thymus of goldfish (*Carassius auratus*) (Ottaviani et al., 1995). In addition, the expression of some immune genes in the central nervous system has been reported, and this suggests a potential cross-interaction between brain immune and neuroendocrine systems (Metz et al., 2006). Assuming that brain and pituitary are the hierarchical onset organs of the stress reaction (Cerdá-Reverter and Canosa, 2009), other central interactions have been shown to occur at brain and pituitary level, particularly the cortisol feed-back interaction via GR (Gorissen and Flik, 2016).

Vaccination is the most effective method used nowadays in aquaculture to prevent diseases caused by pathogens (Plant and LaPatra, 2011). Available data indicates that 2 h after *Vibrio anguillarum* bacterin exposure, the expression of both pro- and anti-inflammatory genes increase in gilthead seabream (*Sparus aurata*) head kidney primary cell culture (Khansari et al., 2017). Moreover, vaccination by immersion leads to alteration of some immune genes including complement *c3*, tumor necrosis factor alpha (*tnfα*), lysozyme (*lys*) *or* transforming-growth factor beta (*tgfβ*) in seabream mucosal tissues such as skin and gut (Khansari et al., 2018). Therefore, these previous results demonstrate that a non-specific immune response is elicited in immune tissues of fish shortly after vaccination. Also, serum or tissue antibodies such as immunoglobulin M and immunoglobulin T will increase at long-term after vaccination (Lamers et al., 1985; Mutoloki et al., 2015), together with some specific immune responses, thus contributing to the increased survival rate when fish are challenged a second time with a pathogen (Rodgers, 1990; Figueras et al., 1998). Similarly, the phagocytic activity of head kidney leucocytes isolated from turbot (*Scophthalmus maximus* L.) enhanced at 7 days post vaccination, and such increase lasted as long as 42 days (Figueras et al., 1998).

There are several vaccine delivery methods, including oral, immersion and injection, of which injection often shows better protection (Plant and LaPatra, 2011). However, the injection procedure can produce adverse reactions due to stress (Hastein et al., 2005), and this unavoidable stress is associated with short-term increase of plasma cortisol (Funk et al., 2004; Skinner et al., 2010). Work on stress or immune effects of vaccine delivered by intraperitoneal injection has been previously reported and shown to be dose and temperature dependent (Martínez et al., 2018; Oyarzún et al., 2019). Nevertheless, few data is available regarding the effects of vaccine on the Hypothalamus-Pituitary-Interrenal (HPI) axis at the brain and pituitary level. In a previous study of our research group, it has been shown that bacterin could elicit immune responses in cultured pituitary cells of rainbow trout (*Oncorhynchus mykiss*) (Liu et al., 2019), and so did when adding medium from cultured spleen cells to pituitary tissue preparations (Liu et al., 2019).

Taking all the above into consideration, the goal of the present study was to investigate the effect of vaccine in both brain and pituitary through different vaccination routes. We hypothesized that: (1) bath vaccination might evoke a significant stress response of the central neuroendocrine organs of fish; and (2) the bacterin vaccines can induce both a stress and immune response in brain and pituitary. To test these hypotheses, two experiments were performed: In the first experiment, *S. aurata* individuals were vaccinated by bath vaccine, subjected to air exposure stress or subjected to both (vaccine and air exposure). Plasma cortisol content as well as gene transcripts relevant to stress responses, specifically, *crh*, *crhbp*, *pomca*, *pomcb, gr, trh, gh, prl, sl1, and sl2* were tested in the pituitary and/or brain at 1, 6, and 24 h post treatments. In the second experiment, we tested whether a vaccine administered through intraperitoneal injection was able to elicit a central stress response. It was also evaluated whether brain and pituitary showed a significant immune response. As fish were taken out of the water for the injection, the responses to vaccination were tested against the air-exposed mock group, thus allowing consistent comparisons with the air exposure group from the first experiment. The air exposure stressor was selected for two reasons. One, because this is a previously used and validated type of stressor related to hypoxia or anoxia experiments (Skrzynska et al., 2018). Second, because we wanted to differentiate the response of the vaccine itself compared to the response induced by a non-biotic physical stressor.

## Materials and Methods

### Fish Husbandry and Experimental Design

Two batches of gilthead seabream (110.8 ± 13.4 g and 285.6 ± 30.2 g) were transported in March and September 2017, respectively, from *Aquacultura Els Alfacs* (Tarragona, north-east Spain). Fish were stocked in the indoor water circular tanks (2000 L) 20 days at the Universitat Autonoma de Barcelona fish facility (AQUAB), under a 12L: 12D photoperiod, 21.4 ± 0.6°C temperature, and they were fed with a commercial diet (Skretting) once per day at a maintenance ration (1.5% body weight). During this period, no clinical signals of disease, malformation or injuries were observed, nor altered behavior. Water parameters including pH, NO_2_, NO_3_, NH_4_/NH_3_, temperature, and salinity were monitored every day. All experimental procedures were submitted by the Ethical Committee of the Universitat Autonoma de Barcelona (CEEAH), in accordance with the international European Guiding Principles for Biomedical Research Involving Animals (EU2010/63) and authorized by the regional authority (Generalitat de Catalunya Procedure Ref. 10208).

### Vaccines and Sample Collection

#### Experiment 1

Seabream were vaccinated with ICTHIOVAC ^R^VR by immersion according to guidelines recommended by the company (HIPRA). ICTHIOVAC ^R^VR (HIPRA) is an inactivated commercial vaccine which is suitable for immersion delivery. The composition consists of inactivated *V. anguillarum*, serotype O1, O2α, and O2β with relative percent survival RPS ≥ 60%, presenting all pathogenic serotypes of the bacterium, including the serogroup O2α that is the most pathogenic serogroup of the bacterium (Frans et al., 2011). The second stressor, air exposure, consisted in 3 min out of the water. To this end, four groups of fish (*n* = 18 fish per group) were used for the experiment: (i) control group, fish treated with water free-vaccine in bucket, (ii) group treated with the vaccine, (iii) group subjected to air exposure during 3 min, and (iv) group exposed to both air exposure and vaccine. There were two replicate tanks in each group. It is worth to mention that vaccination was performed 24 h before air-exposure stress since the preliminary result in systemic immune organs did not show any significant alteration by vaccine at early time of vaccination (data not shown). Fish were sampled after 1, 6 and 24 h. Fish were anesthetized by an overdose of tricaine methanosulphonate (MS222) and the blood from each fish was quickly collected from the caudal vein by using a heparinized 2 mL syringe. After fast blood collection, the pituitary gland and brain of each fish were excised, immediately frozen in liquid nitrogen and stored under -80°C until use.

#### Experiment 2

Before the start of the experiment, a total of 36 fish were randomly divided into 2 groups (with two replicate tanks per group) as for mock injection and vaccination, and these fish were acclimatized for another 5 days in 200 L water circular tanks. During this period, water parameters and rearing conditions were kept the same as mentioned above. After 24 h fasting, all fish were slightly anesthetized by MS222 (0,1 g/L) Sigma-Aldrich, United States), and then they were quickly intraperitoneally injected with 1 mL sterilized PBS or 1 mL ICHTHIO-LG for the mock injection or vaccination groups, respectively. After the injection, fish were immediately returned to the corresponding experimental tanks. The whole operation lasted less than 3 min. Fish from both mock injection and vaccination groups were sampled at 1, 6, and 24 h post injection, and blood, pituitary and brain of 6 fish from each group at each sampling time point were collected. In brief, fish was anesthetized by an overdose of MS222, the blood of each fish was quickly collected from the caudal vein by using a 5 mL syringe, which was pre-rinsed with lithium heparin (Deltalab, Spain), and then transferred to a clean tube with one drop of lithium heparin. After fast blood collection, the pituitary gland and brain of each fish were excised, immediately frozen in liquid nitrogen and stored under -80^o^C until use. ICTHIO-LG *Lactococosis* (HIPRA, Spain) is a vaccine obtained from inactivated *Lactococcus garvieae*, a pathogenic agent for both cultured freshwater and marine fish at water temperature above 15°C. The composition consists of inactivated *L. garvieae* with RPS > 75%.

### Plasma Isolation and Test of Biochemical Parameters

Plasma was separated by blood centrifugation at 1500 ×*g* for 10 min at 4°C. Then the isolated plasma of each fish was transferred to a clean tube and stored at -20°C until biochemical analyses. Plasma CRH and ACTH concentration were detected by using Fish CRH ELISA Kit (Cat: CK-E93386F, Yuan Ye Biotechnology, Shanghai, China) and Fish ACTH ELISA Kit (Cat: CK-E 93337F, Yuan Ye Biotechnology, Shanghai, China) according to manufacturer instructions, respectively. These Elisa kits used in the present study showed sensitivities of about 1.0 pg/mL. The intraassay coefficients of variation were <15% for both two kits. Seabream plasma cortisol levels were measured by radioimmunoassay (RIA) as described by Rotllant et al., 2006 (antibody from MO bio-medical LLC, United States, final dilution 1:4500, lower detection limit of the cortisol assay: 0.16 ng/mL, 100% antibody cross-reactivity with cortisol).

### Total RNA Extraction and Reverse Transcription Quantitative Real-Time PCR (RT-qPCR)

Total RNA of each tissue was extracted according to the manufacturer’s instructions with TRI reagent (Sigma-Aldrich, United States). The RNA concentration of RNA (260 nm) and the purity ratio (A260/A280) was measured with NanoDrop 2000 Spectrophotometer (Thermo Fisher Scientific Inc., United States). First-strand cDNA of each sample was synthesized from 1 μg total RNA by using High-Capacity cDNA Reverse Transcription Kit (Applied Biosystems, United States) according to the user’s manual.

RT-qPCR was performed using iTaq^TM^ Universal SYBR^®^Green Supermix (Bio-Rad, United States) in a CFX Touch^TM^ Real-Time PCR Detection System (Bio-Rad, United States). In brief, a volume of 10 μL containing 0.4 μM of each upstream and downstream primer (Table 1), 2 μL of cDNA product, 2.6 μl of MQ water, and 5 μL of 2 × iTaq Universal SYBR green Supermix were used for the RT-qPCR reaction. The cycling condition consisted of an initial denaturation cycle for 5 min at 95°C, 40 cycles of 15 s at 95°C, 30 s at 60°C. A melting curve analysis was carried out after the completion of RT-qPCR to verify no non-specific amplification. The reference genes 18S and RPL27 were used for normalization. The quantification was performed according to Pfaffl method (Pfaffl, 2001) and corrected for the efficiency of each primer set. Value for each experimental condition was expressed as normalized relative expression, calculated in relation to the values of control group and normalized against those of the reference gene 18S. The amplification efficiency and product size are listed in Table 1. Six biological replicates with two technical replicates were performed for the qPCR analysis.

**Table 1:** Primer information used in the present study.

genes	Primer sequence (5′-3′)	Accession number	Product size (bp)	Efficiency
*Il1β*	F: TCAGCACCGCAGAAGAAAAC R: TAACACTCTCCACCCTCCAC	AJ277166.2	115	1.97
*cox2*	F: GAGTACTGGAAGCCGAGCAC R: GATATCACTGCCGCCTGAGT	AM296029.1	192	1.89
*tnfα*	F: TCGTTCAGAGTCTCCTGCAG R:AAGAATTCTTAAAGTGCAAACACACCAAA	AJ413189.2	320	2.24
*c3*	F: GTTCCACAACAACCCACAGC R:ACATACGCCATCCCATCCAC	HM543456.1	183	1.91
*lys*	F: TCATCGCTGCCATCATCTCC R:TGTTCCTCACTGTCCCATGC	AM749959.1	154	2.08
*tgfβ1*	F: AGACCCTTCAGAACTGGCTC R:ACTGCTTTGTCTCCCCTACC	AF424703.1	145	1.9
*il10*	F: GATCTGCTGGATGGACTGC R: GAGCGTGGAGGAATCTTTCAA	JX976621.1	154	2.02
*il6*	F: ATCCCCTCACTTCCAGCAGA R: GCTCTTCGGCTCCTCTTTCT	EU244588.1	129	2.04
*hsp70*	F: AGGTTGGGTCTGAAAGGAAC R: TGAACTCTGCGATGAAGTGG	EU805481.1	174	1.96
*hsp90*	F: GTGGATTCTGAGGACCTGCC R: GAGAGTCTTCGTGGATGCCC	DQ524994.1	196	1.96
*mt*	F: CTCTAAGACTGGAACCTG R: GGGCAGCATGAGCAGGAG	U93206.1	93	2.07
*crh*	F: ATGGAGAGGGGAAGGAGGT R: ATCTTTGGCGGACTGGAAA	KC195964.1	176	1.86
*trh*	F: GAAACGCTTTTGGGATAACTCC R: CGGCGTGACTCTTGTTTATGTT	KC196277.1	131	2.24
*gh*	F: CGTCTCTTCTCAGCCGAT R: GCTGGTCCTCCGTCTGC	U01301.1	131	1.79
*prl*	F: TGACATCGGCGAGGACAACATT R: CGGCAGCGGAGGACTTTCAG	AJ509807.1	111	1.84
*crhbp*	F: GCAGCTTCTCCATCATCTACC R: ACGTGTCGATACCGCTTCC	KC195965.1	147	1.95
*pomca*	F: AGCCAGAAGAGAGAGCAGTGAT R: ATCGGGTCAGAAAACACTCA	HM584909.1	120	1.92
*pomcb*	F: AGCTCGCCAGTGAGCTGT R: CCTCCTGCATCACTTCCTG	HM584910.1	81	2.07
*gr*	F: TGCTGGCGGAGATCATCACCA R:GCAGGCCAAGCGAAGGCTTA	DQ486890.1	182	2.01
*18s*	F: ACCAGACAAATCGCTCCACC R: AGGAATTGACGGGAAGGGCAC	AY587263.1	172	2.02
*rpl27*	F: AAGAGGAACACAACTCACTGCCCCAC R: GCTTGCCTTTGCCCAGAACTTTGTAG	AY188520.1	160	2.01


### Statistics

For the first experiment the statistical package for social science (SPSS, v20) software was used for the analysis. The Generalized Linear Model (GzLM) was utilized considering the stressors and time dynamics as a two between-subjects factor. This model is a more flexible statistical tool than the standard general linear model (GLM) in terms of types of distribution and different covariance structure of the repeated measures does not require homogeneity of variance and it admits missing values. After the main analysis, appropriate pairwise comparisons were carried out. In the second experiment, we used either one-way ANOVA followed by Fisher’s LSD post-hot test, or unpaired student’s *t*-test if the equal variances were not assumed. Differences among groups were considered significant when *P* < 0.05. All results were expressed as mean ± SEM.

## Results

### Brain and Pituitary Stress Response to Bath Vaccine

**FIGURE 1:**
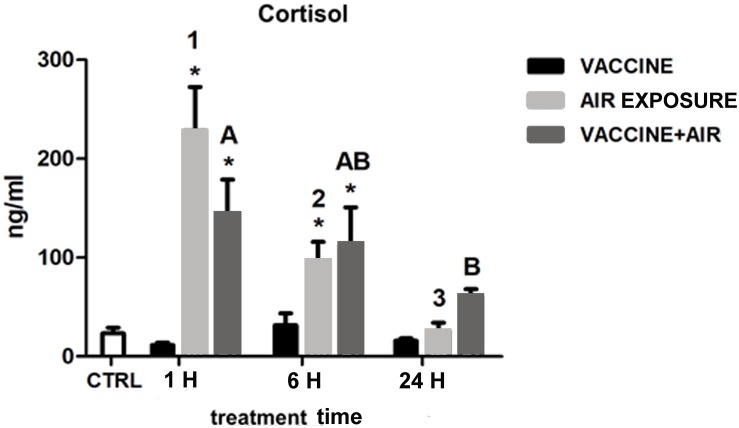
Plasma cortisol concentration in gilthead seabream (*Sparus aurata* L.) at 1, 6, and 24 h post challenge after air exposure and *Vibrio* vaccine. Data are presented as mean ± SEM. Significant differences are indicated by numbers in air exposure group and by capital letters in vaccine plus air exposure group. Asterisk (^∗^) indicates significant difference vs. control and the absence of a symbol indicates no difference (*P* < 0.05; General-linear-Model test was performed for multiple comparison).

Figure 1 shows the levels of plasma cortisol after both stressors and its combination at the respective time points. Cortisol did not show any significant response after bath vaccine treatment compared to air exposure stress, which showed a classical acute response dynamics with a peak at 1 h, still significantly higher at 6 h, followed by further recovery of basal values at 24 h. The dynamics of the vaccine plus air exposure was similar to that observed after air exposure, although the recovery took place later on, indicating that the air exposure stressor was predominant in the cortisol response.

**FIGURE 2:**
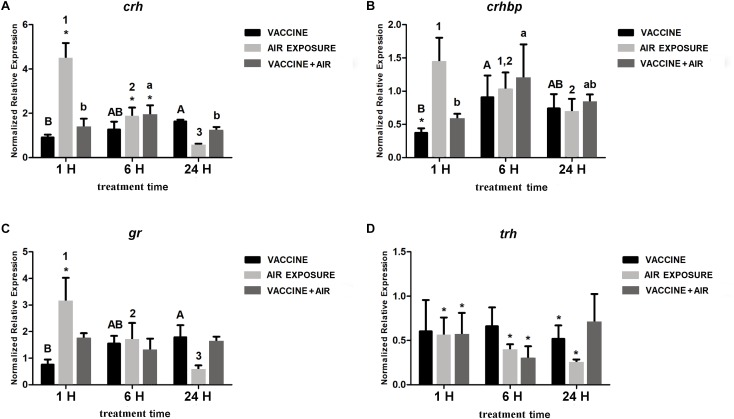
qPCR quantification of specific mRNA accumulation in gilthead seabream (*Sparus aurata* L.) brain at 1, 6, and 24 h post challenge with *Vibrio anguillarum* exposure and air exposure. **(A)** *crh*; **(B)** *crhbp*; **(C)** *gr*; and **(D)** *trh* were shown as mRNA relative abundance. Data are presented as mean ± SEM. Significant differences are indicated by capital letters in vaccine group, by numbers in air exposure group, and by lowercase letters vaccine plus air exposure. Asterisk (^∗^) indicates significant difference versus control and the absence of a symbol indicates no difference (*P* < 0.05; General-linear-Model test was performed for multiple comparison).

Regarding the response of the analyzed stress-related genes in the brain, no relevant changes were observed after bath vaccine treatment, except for a decrease of *crhbp* at 1 h, whereas air exposure showed significant increases in *crh, crhbp*, and *gr* and a significant down-regulation of *trh*. When both stressors were applied, *trh* was maintained down-regulated at 6 h and only *crhbp* increased significantly (Figure 2).

**FIGURE 3:**
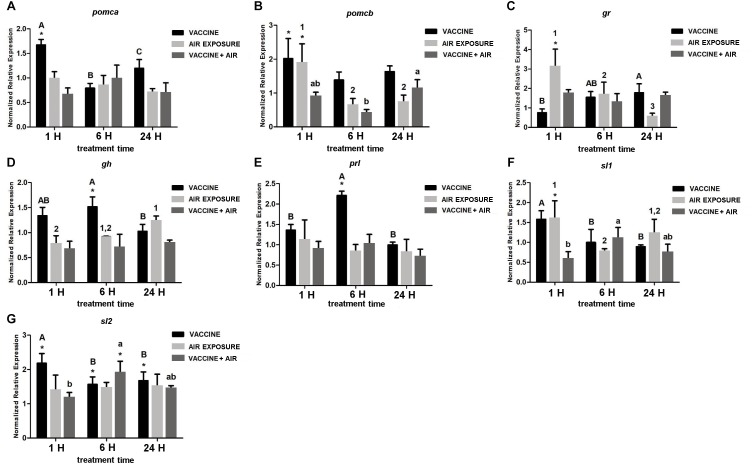
qPCR quantification of specific mRNA accumulation in gilthead seabream (*Sparus aurata* L.) pituitary at 1, 6, and 24 h post challenge with air exposure and *Vibrio anguillarum* exposure. **(A)** *pomca*; **(B)** *pomcb*; **(C)** *gr*; **(D)** *gh*; **(E)** *prl*; **(F)** *sl1*; and **(G)** *sl2* were shown as mRNA relative abundance. Data are presented as mean ± SEM. Significant differences are indicated by capital letters in vaccine group, by numbers in air exposure group, and by lowercase letters vaccine plus air exposure. Asterisk (^∗^) indicates significant difference versus control and the absence of a symbol indicates no difference (*P* < 0.05; General-linear-Model test was performed for multiple comparison).

In the pituitary, bath vaccination showed a differential induction of *pomc* genes at short time (1 h) whereas at 6 h *prl* and *gh* showed significant increases. Air exposure increased the expression of *pomcb, gr* and *sl1* at 1 h, and *gr* at 6 h. After applying both stressors only slight changes were detected as for the reductions of *pomcb* at 6 h and *sl1* at 1 h, and the increase of *sl1* at 6 h (Figure 3).

### Brain and Pituitary Stress and Immune Responses to Injected Vaccine

**FIGURE 4:**
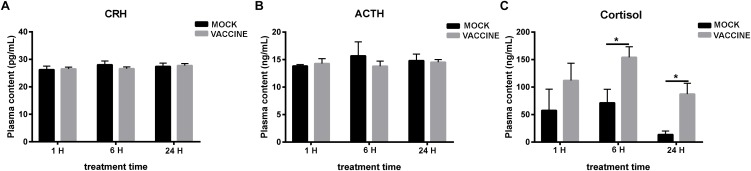
Plasma CRH **(A)**, ACTH **(B)**, and cortisol **(C)** concentration in gilthead seabream (*Sparus aurata* L.) after *Lactococcus* bacterin or mock injection. Asterisk (^∗^) indicates significant difference between mock and vaccination groups of each time point (*P* < 0.05).

Plasma cortisol values significantly raised by 2.2- and 6.4-fold compared to the corresponding mock groups at 6 and 24 h post injection, respectively (*P* < 0.05). The differential cortisol increase of the vaccine-injected fish compared to the mock-injected fish was apparent at all time points. Regarding time course, both injected vaccine and mock groups presented the same cortisol dynamics, i.e., increases at 1 and 6 h and recovery at 24 h. However, the vaccinated group showed higher levels at either time compared to mock-injected group. The vaccine injected group also showed higher resistance to recovery at 24 h. Regarding CRH or ACTH, no alteration of plasma content in either group (mock or vaccination) was observed in none of different time points assessed 1, 6, and 24 h post injection (Figure 4).

**FIGURE 5:**
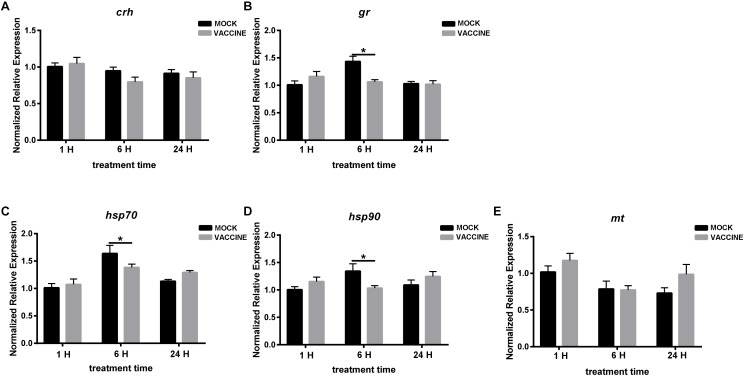
mRNA expression of genes involved in the HPI axis in the brain of gilthead seabream (*Sparus aurata* L.) after *Lactococcus* bacterin or mock injection. **(A)** *crh*; **(B)** *gr*; **(C)** *hsp70*; **(D)** *hsp90*; and **(E)** *mt*. Asterisk (^∗^) indicates significant difference between mock and vaccination groups of each time point (*P* < 0.05).

In the second experiment, the expression of *crh* was almost unchanged at 1, 6, and 24 h post vaccination. Transcript of *gr* was not altered at 1 or 24 h, while there was a slight but significant up-regulation at 6 h post vaccination in the mock group. A similar trend can be observed in the heat shock proteins (HSP) *hsp70* and *hsp90* in which a significant increase was also observed at the same time point (6 h). As a whole, few changes were observed in brain genes, and the changes were higher in mock-injected fish than in vaccine-injected fish (Figure 5).

**FIGURE 6:**
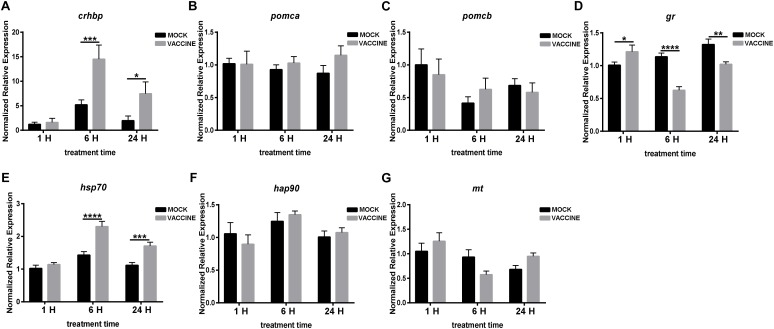
mRNA expression of genes involved in the HPI axis in the pituitary of gilthead seabream (*Sparus aurata* L.) after *Lactococcus* bacterin or mock injection. **(A)** *crhbp*, **(B)** *pomca*, **(C)** *pomcb*; **(D)** *gr*; **(E)** *hsp70*; **(F)** *hsp90*; and **(G)** *mt*. Asterisk (^∗^) indicates significant difference between mock and vaccination groups of each time point (*P* < 0.05).

Similar than with the bath vaccine, the expression of stress genes in the pituitary showed a different pattern in which one gene, CRH binding protein (*crhbp*), substantially increased its expression (up to 15 fold at 6 h or up to 7 fold after 24 h) after vaccine injection. These increases contrast with the mock injected fish in which the increase was moderate (between 3 and 5 fold), though showing the same dynamics. A similar significant trend, but more moderate (over two fold increases), was observed for *hsp70* but not for *hsp90*. The rest of the genes assessed, although showing some variations, did not change significantly their expression except for *gr* in which a significant down-regulation was observed at 6 h (Figure 6).

### mRNA Expression of Immune Genes in Brain and Pituitary

**FIGURE 7:**
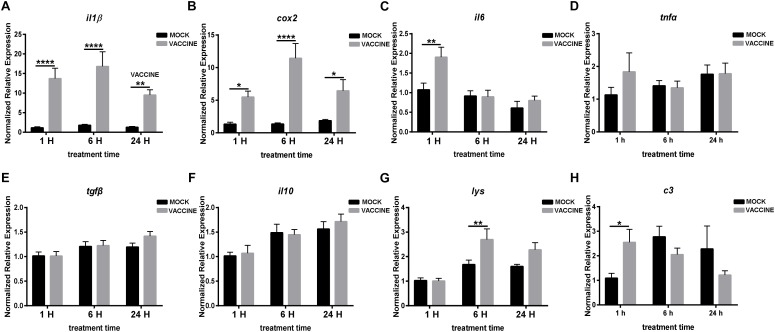
RNA expression of pro-inflammatory genes *il1β* **(A)**, *cox2* **(B)**, *il6* **(C)**, *tnfα* **(D)**, anti-inflammatory genes *tgfβ* **(E)**, *il10* **(F)** and some other immune components *lys* **(G)** and *c3* **(H)** in the brain of gilthead seabream (*Sparus aurata* L.) after *Lactococcus* bacterin or mock injection. Asterisk (^∗^) indicates significant difference between mock and vaccination groups of each time point (*P* < 0.05).

In the second experiment it was intended to determine whether an injected vaccine induced immune gene expression changes in brain and pituitary other than in stress-related genes. The results showed a very clear picture as not only some cytokines increased their expression but the level of induction was very strong. Thus, the main pro-inflammatory cytokine *il1β* dramatically raised in the vaccination group at all three time points: 1, 6, and 24 h, by 12.0, 9.36, and 7.44 fold, respectively. Similarly, *cox2* was significantly up-regulated by 5.25, 7.77, and 3.46 fold in the vaccination group at 1, 6, and 24 h post injection, respectively. The expression level of both *il1β* and *cox2* peaked at 6 h post vaccination group. Other pro-inflammatory gene transcripts such as *il6* raised significantly only 1.7 fold at 1 h post vaccination (*P* < 0.01), and *tnfα* was kept almost unchanged. Differently from the pro-inflammatory genes, the classical anti-inflammatory genes *tgfβ* and *il10* showed no significant alteration in the vaccinated groups when compared with the corresponding mock injection groups. Nevertheless, the mean values showed a non-significant but apparent increasing trend (Figure 7). The expression of *lysozyme* gene (*lys*) significantly increased in the brain of seabream at 6 h post vaccination, although with just 1.61 fold, and a moderate raise was also observed at 24 h post injection. The transcript for the complement C3 component gene (c3) showed a significant increase at 1 h post vaccination, by 2.35 fold; however, it decreased at 6 and 24 h after vaccination when compared with the corresponding mock injection groups (Figure 7).

**FIGURE 8:**
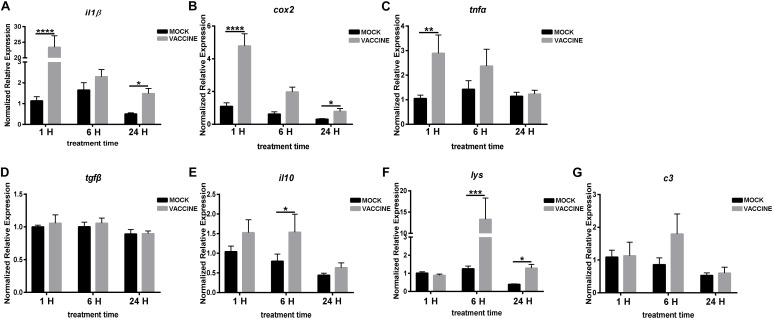
mRNA expression of pro-inflammatory genes *il1β* **(A)**, *cox2* **(B)**, *tnfα* **(C)**, anti-inflammatory genes *tgfβ* **(D)**, *il10* **(E)** and other soluble immune regulators *lys* **(F)**, *c3* **(G)** in the pituitary of gilthead seabream (*Sparus aurata* L.) after *Lactococcus* bacterin or mock injection. Asterisk (^∗^) indicates significant difference between mock and vaccination groups of each time point (*P* < 0.05).

Regarding pituitary, the expression of genes related to the immune responses are shown in Figure 8. The expression of pro-inflammatory gene *il1β* sharply and strongly increased at the three time points post vaccination, and it was significantly up-regulated by 20.65 and 2.94 fold at 1 and 24 h, respectively. A similar alteration trend was observed for the expression of *cox2* after vaccination, however, with less intensity. The *cox2* transcript raised by 4.37, 3.16, and 2.52 fold in 1, 6, and 24 h vaccination groups, respectively, but significance was observed only at 1 and 6 h time points. The *tnfα* transcript was up-regulated at 1 and 6 h post vaccination, and the significance was only observed in the early phase of vaccination (1 h), by 2.74 fold. The anti-inflammatory gene *tgfβ* showed no alteration after vaccination, and *il10* presented a raising trend, although significant induction was only observed at 6 h. Compared to the mock injection, *lys* showed comparable levels at 1 h post injection, while it was distinctly up-regulated by 10.59 and 3.25 folds at 6 and 24 h, respectively.

## Discussion

### Brain and Pituitary Stress Response to Vaccines

The combined results from the two vaccination experiments indicate that fish perceives the vaccine as a stressor but at a limited extent. Thus, bath vaccine did not induce plasma cortisol rise, while injection vaccine did produce a differential cortisol response compared to mock injection. In addition, neither plasma CRH nor ACTH values showed important alterations after vaccine injection. Therefore, in terms of plasma hormones, it seems that fish would not perceive vaccines as primary stressors stimulating the hierarchical activation of HPI axis, although it would indirectly activate cortisol release in the case of vaccine injection linked to the air exposition period during the injection procedure. In both experiments cortisol presented an acute response dynamics, peaking at 1 or 6 h and recovering at 24 h, in agreement with the studies previously reported for this species after subjecting seabream to acute stressors such as air exposure (Arends et al., 1999; Skrzynska et al., 2018). Similar increases of cortisol concentration in rainbow trout treated by vaccine injection have been previously reported as well (Funk et al., 2004; Skinner et al., 2010). However, in other works in which higher doses of bacteria were administered to *Eleginops maclovinus* or *S. maximus*, increases of cortisol lasted for 7 days or even longer after injection (Rodríguez-Quiroga et al., 2017; Oyarzún et al., 2019).

Our results also suggest that vaccines do not clearly activate the response of brain stress genes during the first hours. Thus, neither *crh* nor *crhbp* or *gr* showed relevant modulation after bath vaccine and only slight changes were observed in *hsp* and *gr* after injection. Therefore, this suggests that vaccine did not activate the central stress gene response unless a physical stressor (air exposure) was included, as observed in the vaccine plus air exposure groups. This agrees with the previously reported response of *S. aurata* to different stressors (Skrzynska et al., 2018).

On the contrary, in the pituitary, vaccine did induce the gene expression of stress-related hormones like *prl, gh* at 6 h and *pomca* and *pomcb* peptides at 24 h. Therefore, it seems that the pituitary was more sensitive than brain to immune stimulation, although at later time points (6 and 24 h). This may indicate, other than a higher sensitivity, that the pituitary stimulation could be not a direct effect, but resulting from the interaction through biological messengers such as cytokines. This would be supported by the fact that both brain and pituitary showed a robust pro-inflammatory cytokine response to vaccine (see Figure 7, 8). At this point, the research has not gone further as receptors for *pomc* have not yet been cloned in seabream, although attempts have been made by several laboratories. The results for *trh* seem to follow a similar trend than *crh*, i.e., reduced activation of the stress axis, showing a small variation as a response to bath vaccine. At 1 h, levels were higher than at 6 h which matches with previous results of Ruiz-Jarabo et al. (2017) and Skrzynska et al. (2018). This response could also be related to an inhibition of the thyroid hormone axis modulating energetic responses, thus contributing to save energy resources. This moderate response is also linked to the expression of both *pomc* genes. Thus, *pomcb* showed a decrease of its expression at 6 h as *trh*, whereas *pomca* and *crh* maintained unaltered levels.

It is worth to note that vaccination caused a raise of cortisol together with some alteration of both *hsp* and *mt* in brain and pituitary. Metallothioneins, similarly than HSP are involved in stress response, and their expression can be induced by cortisol in fish (Hyllner et al., 1989) as a result of both abiotic and biotic stressors (Iwama et al., 1999; Roberts et al., 2010; Yamashita et al., 2010). In addition, our recent data in skin mucus showed both an increment of cortisol induced by *V. anguillarum* vaccine and also a significant rise of *hsp70* (Khansari et al., 2018). During stress, global RNA translation is supposed to be reduced to save energy, while a selective translation is up-regulated, which facilitates coping with challenges (Holcik and Sonenberg, 2005; Tort and Teles, 2011). Thus, after vaccination, a decreased expression of some stress genes together with the enhancement of other key response genes might be associated to energy savings, and thus protective immunity responses could be maintained (Pulendran and Ahmed, 2011).

Besides the role of GR in mediating the glucocorticoid effect of cortisol in target tissues, some other factors such as CRH and CRHBP could serve as potential feedback agents in the HPI axis. CRH is an ancient stress neuropeptide which is essential for facilitating the adaptive response to environmental stressors (Denver, 1999). Binding of CRH with its receptors in pituitary cells stimulates ACTH production. With a high affinity to CRH, CRHBP can sequester CRH in the circulation and thus modulate its bioavailability (Huising et al., 2004). Normally, a large proportion of total circulating CRH is complexed with CRHBP, and therefore the availability for receptor activation is low (Behan et al., 1997). Thus, CRHBP would act as another potential negative feedback agent of HPI axis as suggested by the up-regulation of *crhbp* in the pituitary of seabream after vaccination. This agrees with such a proposed role of CRHBP during acute stress in *S. aurata* in previous works (Martos-Sitcha et al., 2014).

Combining the results of *gr* expression and *crhbp* with the plasma cortisol content, we could speculate that after cortisol increase, up-regulation of *crhbp* and down-regulation of *gr* constitute two feedback factors of HPI axis. They would indirectly inhibit the bioavailability of circulating CRH and then suppress the new production of cortisol, reducing the binding of cortisol with GR and finally leading to the descent bioactivity of cortisol. Besides, induction of inflammation may help to eliminate potential invading pathogens and dead cells. On the other hand, prolonged hyperactivation of the immune response may be detrimental and therefore anti-inflammatory cytokines would help to regulate this activation process, which matches with the increase of *il10* observed both in brain and pituitary. Thus, the simultaneous alteration of plasma cortisol, decreased expression of *gr*, up-regulated expression of *crhbp* and pro-inflammatory genes, and the down-regulation of anti-inflammatory genes could constitute a beneficial picture for homeostasis and recovery of fish.

Overall, in terms of the effect of the vaccine (route of vaccination or the bacterial species) the comparison between both experiments indicates that the stress response to vaccines focuses more in pituitary or head kidney (associated to the increase of cortisol) than in the brain. As mentioned before, while slight changes are recorded in either plasma hormones or genes in brain, in pituitary bath vaccine did modify *pomca, pomcb* expression at 1 h and *pomcb* at 6 h. In addition, *gr* increases at 6 h after bath and decreases after injection vaccination.

### Immune Gene Response in Brain and Pituitary After Vaccine Injection

Immune responses in fish such as inflammatory and antibody response elicited by bacteria or pathogen-associated molecular patterns (PAMPs) including LPS, poly (I:C), can be detected notably in immune organs like spleen and head kidney (Cvitanich et al., 1991; Reyes-Cerpa et al., 2012; Martínez et al., 2018; Sheng et al., 2019). A relevant result of the present study is the strong immune response occurring both in brain and pituitary after vaccination regardless the overall stress response. Thus, very significant alterations of *il1β, cox2, lys, and c3* were observed in the brain and pituitary of vaccinated seabream. IL1β is one of the first cytokines produced at the inflammation site that contributes to induce the expression of other cytokines including TNFα, IL1α, IL6, IL8, COX2, MCP1 (Weber et al., 2010; Zou and Secombes, 2016). Cox2 is a potent mediator of inflammation encoding a prostaglandin-endoperoxide synthase 2, which is a rate limiting enzyme for formation of prostaglandins (PG) functioning under a wide variety of challenging conditions (Smith et al., 1996; Ricciotti and FitzGerald, 2011). The significant raise of pro-inflammatory signaling genes observed in brain and pituitary suggests that vaccines induced inflammation in these two tissues. It can also be speculated that stimulation of cortisol by the vaccine may be associated with an interaction of *il1β* expression at the pituitary, as previously proposed (see Tort and Teles, 2011). Moreover, the increased expression of *c3*, responsible for the complement protein C3, and even more the dramatic raise of *lys*, responsible for the bacteriolytic protein lysozyme (Sunyer et al., 1997; Hernández and Tort, 2003; Saurabh and Sahoo, 2008), indicates an effective activation of innate immune responses in the central neuroendocrine tissues after the intraperitoneal vaccination. The combination of the alteration of inflammatory genes plus the increase of the immune innate genes supports the occurrence of a significant immune response in the pituitary.

There is not precisely known what are the precise mechanisms of interaction between hormone elements and immune agents. Normally, due to the protection of the blood brain barrier, pathogens can hardly access to the brain or pituitary. However, some mediators such as cytokines can play a role of connecting antigens and response (Banks et al., 1995). In our previous works we observed that both the medium from the *in vitro* cultured spleen and recombinant IL1β presented a significant effect on the *in vitro* immune response of trout pituitary (Liu et al., 2019). Thus, the immune response in the central neuroendocrine system might be regulated by some mediators produced and released into the bloodstream by lymphoid organs as a response to the bacterin delivered by intraperitoneal injection. Further studies will be necessary to precise the mechanisms that can confirm this hypothesis.

Although both brain and pituitary present a robust immune reaction, it is worth noting that the expression trends of immune genes are different between these organs, and this may be related to the respective tissue architecture. Brain is constituted by neurons and also glia which account for an abundant portion of the brain cell population, acting as the primary resident macrophages to elicit both innate and adaptive immune responses (Yang et al., 2010; Schwartz et al., 2013; Lenz and Nelson, 2018). Thus, while pituitary have endocrine cells as the predominant population, just some stellate cells are hypothetically the functional immune cells (Glennon et al., 2015). In brain, the participation of glial cells in immune response could be quantitatively rather higher. Therefore, we can hypothesize that alterations in the transcription levels of immune genes in response to intraperitoneal vaccine injection might result from the different architecture and cell composition of these two organs, thus leading to different signaling elements involved in the immune response. In the case of *L. garvieae*, it is also possible that the strong induction of the pro-inflammatory response could be associated to the pathogenic neurodegenerative effect of the *L. garvieae* that has been shown to produce brain damage in fish (Vendrell et al., 2006). Moreover, although these hypotheses need further investigation, our findings support the fact that fish brain is capable of inducing a strong inflammatory response.

## Conclusion

Vaccination, either via bath or injection did not involve a significant induction of brain-pituitary stress response, although cortisol showed a moderate increase. Other than assuming that such antigen stimulus does not involve a direct and high central perception response, the observed reaction could be also associated to the altered feedback genes of the HPI axis *gr* and *crhbp* that may have played a relevant role in preventing the maintenance of higher cortisol levels in brain and pituitary and therefore also preventing cortisol immunosuppressive consequences. Such a mechanism could modulate the initial stress response and the pleiotropic cortisol action, thus helping to prevent the putative suppression of an active immune response in the neuroendocrine centers. Thus, the raise of cortisol caused by the vaccination would not be achieved through the initial activation of the central brain-pituitary axis elements. Besides, a robust immune response was elicited both in brain and pituitary regardless the route of administration, bath or injection, as shown by the up-regulation of cytokines and innate response genes. Thus, results suggest an active and direct immune action of the vaccine components in brain and pituitary tissues uncoupled from the initial stress HPI axis response.

## Data Availability

All datasets for this study are included in the manuscript and the supplementary files.

## Ethics Statement

All experimental procedures were submitted by the Ethical Committee of the Universitat Autonoma de Barcelona (CEEAH), in accordance with the international European Guiding Principles for Biomedical Research Involving Animals (EU2010/63) and authorized by the regional authority (Generalitat de Catalunya Procedure Ref. 10208).

## Author Contributions

LT, JM, and FR-L conceived and designed the experiments. XL, AK, MT, and FR-L performed the experiments. XL, AK, GM-R, LT, and FR-L analyzed the data. YZ, GM-R, JM, and FR-L contributed the reagents and materials. XL, AK, MT, YZ, JM, GM-R, FR-L, and LT contributed to the writing of the manuscript. All authors read, corrected and approved the final manuscripts.

## Conflict of Interest Statement

The authors declare that the research was conducted in the absence of any commercial or financial relationships that could be construed as a potential conflict of interest.
